# Morphological changes in the meibomian glands of patients with phlyctenular keratitis: a multicenter cross-sectional study

**DOI:** 10.1186/s12886-016-0347-5

**Published:** 2016-10-10

**Authors:** Takashi Suzuki, Naoyuki Morishige, Reiko Arita, Shizuka Koh, Tohru Sakimoto, Rika Shirakawa, Kazunori Miyata, Yuichi Ohashi

**Affiliations:** 1 LIME (Lid and Meibomian gland) working group, Tokyo, Japan; 2 Department of Ophthalmology, Ehime University, Graduate School of Medicine, Shitsukawa, Toon, Ehime 791-0295 Japan; 3 Department of Ophthalmology, Yamaguchi University, Graduate School of Medicine, Ube, Japan; 4 Department of Ophthalmology, University of Tokyo School of Medicine, Tokyo, Japan; 5 Department of Ophthalmology, Itoh Clinic, Saitama, Japan; 6 Department of Ophthalmology, Osaka University Graduate School of Medicine, Suita, Japan; 7 Department of Visual Sciences, Division of Ophthalmology, Nihon University School of Medicine, Tokyo, Japan; 8 Miyata Eye Hospital, Miyakonojo, Japan

**Keywords:** Phlyctenular keratitis, Meibomian glands, Meibography, Meibomitis

## Abstract

**Background:**

Phlyctenular keratitis is a hypersensitivity reaction of the cornea, and a complication of eyelid margin disease in children and young adults. In this study, we compared the morphology of the meibomian glands in eyelids between phlyctenular keratitis patients and healthy young adults, using noncontact meibography.

**Methods:**

The study included 16 eyes of 13 patients diagnosed with phlyctenular keratitis and 17 eyes of 17 healthy volunteers. Slit-lamp observations of the cornea and eyelid were performed on all subjects. The morphology of the meibomian glands was scored using non-contact meibography (meiboscore). The meiboscore in worse eye was used in bilateral phlyctenular keratitis.

**Results:**

All eyes with phlyctenular keratitis, but not normal controls, showed corneal nodules, neovascularization, and superficial punctate keratopathy. The mean meiboscore in phlyctenular keratitis patients (upper lid: 2.9 ± 0.3, lower lid: 2.7 ± 0.5) was significantly higher than in controls (upper lid: 0.4 ± 0.6, lower lid: 0.1 ± 0.3).

**Conclusions:**

Noncontact meibography enabled visualization of meibomian gland loss in phlyctenular keratitis patients, suggesting a relationship between abnormalities of the meibomian glands in young individuals and the pathogenesis of phlyctenular keratitis.

**Electronic supplementary material:**

The online version of this article (doi:10.1186/s12886-016-0347-5) contains supplementary material, which is available to authorized users.

## Background

Phlyctenular keratitis is a complication of eyelid margin disease, primarily affecting children and young adults [1, 2]. The characteristic clinical findings in phlyctenular keratitis include inflammatory corneal nodules with vascularization and meibomitis [3]. The pathogenesis of this disease could involve a delayed-type hypersensitivity (DTH) reaction to foreign microbial proteins from organisms such as *Mycobacterium tuberculosis*, *Staphylococcus aureus*, or *Propionibacterium acnes,* which are found at the eyelid margin and in the meibomian gland [3–10]. Indeed, phlyctenular keratitis can be accompanied by meibomitis or blepharitis, and bacteria such as *S. aureus* and *P. acnes* can be detected in eyelid scrapings or in meibum [3, 4, 10]. Meibomitis, which is inflammation of the meibomian glands, causes the glands to be obstructed by thick waxy secretions, and may trigger phlyctenular keratitis [3]. Suzuki et al. proposed a disease subset termed meibomitis-related keratoconjunctivitis (MRKC), ocular surface inflammatory disease associated with meibomitis; the clinical features of MRKC are similar or identical to those of phlyctenular keratitis [7]. Thus, the observation of eyelid and meibomian gland condition in cases of phlyctenular keratitis may help us to understand the pathogenesis of the disease.

Meibomian gland condition is commonly evaluated based on slit-lamp observations of the meibomian orifices, examination of meibomian gland sebum, and meibography, which assesses the structure of the glands by transillumination. However, it is often difficult to observe the condition of the eyelid or of the meibomian glands in children who cannot be secured to a chin-rest. Additionally, conventional meibography and a slit lamp allows observation of only a limited area of the eyelid [11]. Recently, a noninvasive, mobile, pen-shaped meibography system using an infrared light-emitting diode was developed, and was found to be useful in the observation of meibomian gland structure in infants and in patients with severe systemic diseases [12–14].

Because meibomitis is associated with phlyctenular keratitis, abnormalities of the meibomian glands may be involved its pathogenesis. Little is known, however, about the morphology of the meibomian glands in this disease. Herein we report features of patients with phlyctenular keratitis, observed with noncontact meibography, and compare them to those of healthy volunteers. These observations demonstrate the relationship of morphological changes in meibomian glands to phlyctenular keratitis in patients, and suggest its pathogenesis.

## Methods

### Patients

Patients with phlyctenular keratitis and healthy young adults were examined in Ehime University hospital, Yamaguchi University Hospital, Itoh Clinic, Osaka University Hospital, Nihon University Hospital, Miyata Eye Hospital, and Tokyo University Hospital. Diagnosis of phlyctenular keratitis was made based on bacterial culture of eye lids and clinical manifestations such as corneal nodules, their vascularization, and meibomitis, as described previously [3]. Subjects included 13 patients (four males and nine females; mean ± SD age, 13.8 ± 8.6 years) and 17 healthy volunteers (seven males and ten females; mean ± SD age, 13.4 ± 2.7 years).

### Examination

Slit-lamp observations of the cornea and eyelid were performed. The upper and lower eyelids were turned over and the meibomian glands were observed using a mobile pen-shaped meibograph (Meibopen, JFC Sales Plan Co. Ltd, Tokyo, Japan) or another non-contact meibography system (BG-4 M, TOPCON, Tokyo, Japan). Captured pictures were estimated by graders who were masked to the demographics of the subjects. Partial or complete loss of the meibomian glands for each eyelid was graded by meiboscore as reported previously; grade 0 (no loss of meibomian glands), grade 1 (loss of 1/3 of the total area of meibomian glands), grade 2 (area loss between 1/3 and 2/3), and grade 3 (area loss ≥2/3) [15]. Meiboscores for the upper and lower eyelids were summed to obtain a score for each eye [15]. The meiboscore in worse eye was used in bilateral phlyctenular keratitis.

### Statistical analysis

Meiboscores and ages were compared between phlyctenular keratitis patients and healthy volunteers using Mann-Whitney *U*-test (JMP pro 11, SAS Institute Inc., NC). In cases of unilateral phlyctenular keratitis, we compared the meiboscores between the inflamed and non-inflamed eyes using two-tailed Student’s *t*-test. Gender were compared between phlyctenular keratitis patients and healthy volunteers using a chi-square test. A *p* value of <0.05 was considered to indicate statistical significance. Data are shown as means ± SD unless otherwise specified.

## Results

**Fig. 1:**
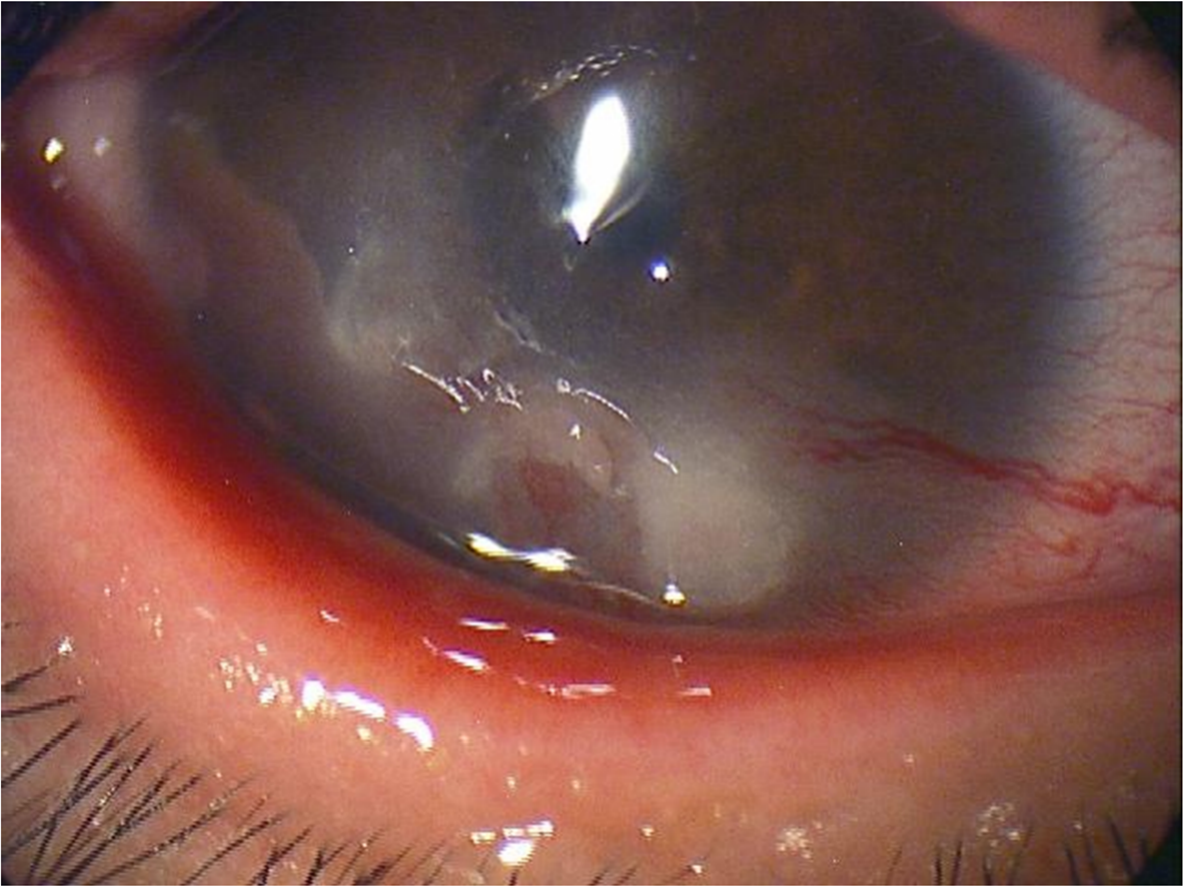
Slit-lamp photograph of case 9 showing corneal nodule, vascularization, and conjunctival injection

A representative clinical photograph of phlyctenular keratitis in a 4-year-old male (patient case 9) with a corneal nodule, neovascularization, hyperemia, and meibomitis in the right eye is shown in Fig. 1. Ocular surface manifestations in 13 patients with phlyctenular keratitis are summarized in Table 1. There were three adults (>20 years), and ten young adults (<16 years). Four of thirteen patients had bilateral phlyctenular keratitis. Inflamed eyes of all patients had corneal nodules, neovascularization, and superficial punctate keratopathy (SPK). Five patients had a history of chalazia. Healthy volunteers showed no clinical features. Gender and age were not significantly different between the phlyctenular keratitis patients and the healthy volunteers (Table 2).

**Table 1:** Summary of 13 patients with phlyctenular keratitis

					Ocular manifestation				Meiboscore		
Case	Gender	Age range of onset (y)	Eye(s)	History of chalazia	Corneal nodule	NV	SPK	Hyperemia	Upper lid	Lower lid	Total
1	F	20–30	OS	+	+	+	+		3	2	5
2	F	20–30	OS	−	+	+	+	+	3	3	6
3	F	10–20	OU	−	+	+	+		3	2	5
4	F	10–20	OU	−	+	+	+	+	3	3	6
5	F	30–40	OU	−	+	+	+	+	3	3	6
6	F	10–20	OS	−	+	+	+	+	2	3	5
7	M	10–20	OD	+	+	+	+	+	3	3	6
8	M	0–10	OS	+	+	+	+	+	3	3	6
9	M	0–10	OD	+	+	+	+	+	3	3	6
10	M	0–10	OS	−	+	+	+	+	3	3	6
11	F	10–20	OS	+	+	+	+	+	3	3	6
12	F	10–20	OD	−	+	+	+	+	3	2	5
13	F	10–20	OU	−	+	+	+	+	3	2	5

*F* female, *M* male, *OD* right eye, *OS* left eye, *OU* both eyes, *NV* corneal neovascularization, *SPK* superficial punctate keratopathy

OU showed identical clinical features

**Table 2:** Clinical features and meiboscores in patients and controls

	Phlyctenular keratitis (*n* = 16)	Controls (*n* = 17)	*P*
Gender (M/F)	4/9	7/10	0.56
Age	13.8 ± 8.6	13.4 ± 2.7	0.56
Meiboscore (Mean ± SD)			
Upper	2.9 ± 0.3	0.4 ± 0.6	<0.0001
Lower	2.7 ± 0.5	0.1 ± 0.3	<0.0001

*F* female, *M* male, *N* number of patients

**Fig. 2:**
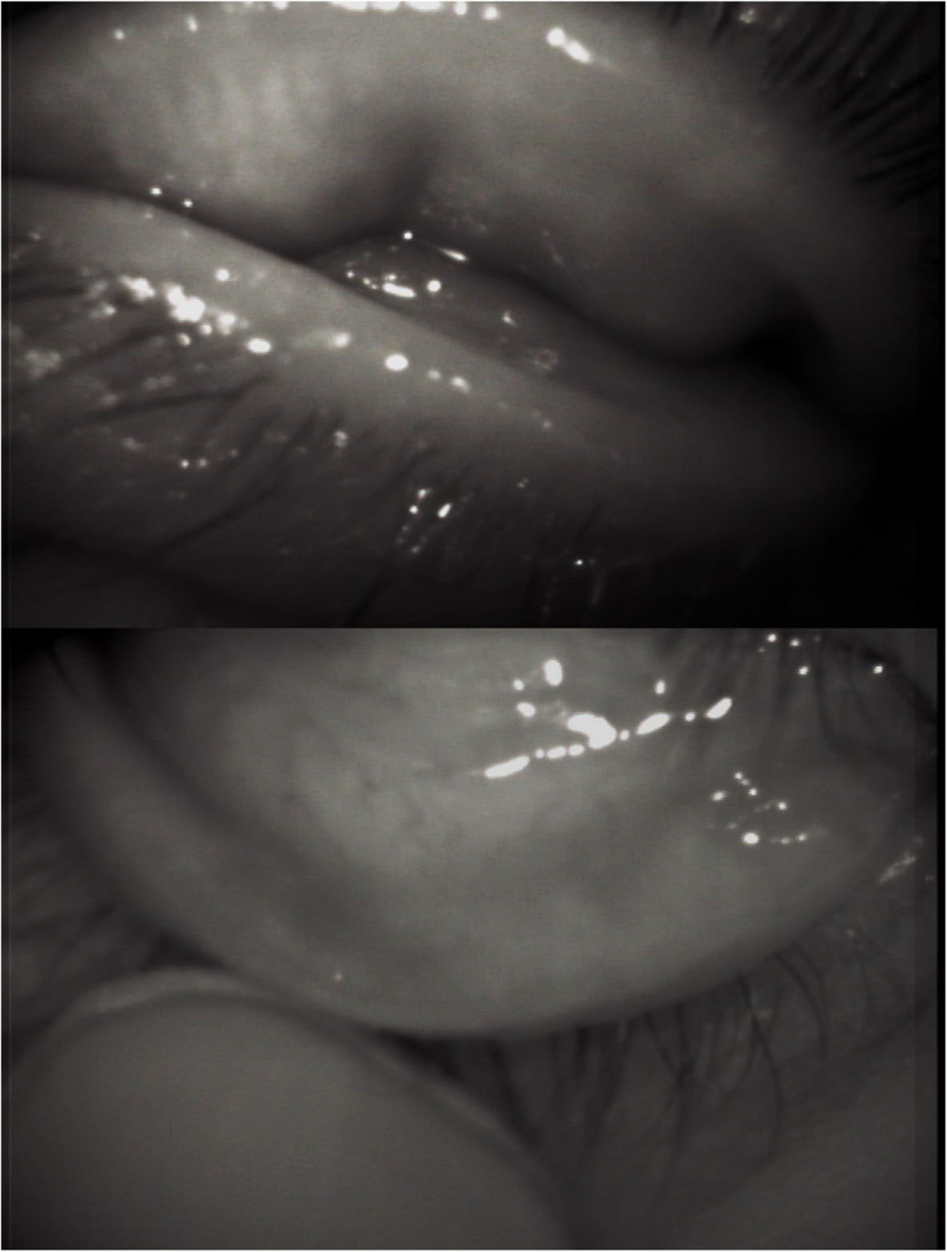
Noncontact meibography imaging of case 9 showing marked loss of the meibomian glands in the upper (*top*) and lower eyelids (*bottom*). Meiboscore (upper lid:3, lower lid:3, total:6)

The morphology of the meibomian glands in the eyelids of 13 phlyctenular keratitis patients (13 eyes) and 17 healthy volunteers (17 eyes) were compared. This demonstrated meibomian gland loss in both the upper and lower lids of the right eye of the patient identified as case 9 (Meiboscore;upper lid:3, lower lid:3, total:6), while this patient’s non-inflamed left eye showed meibomian glands of a normal morphology (Figs. 2a and b). The meiboscores of the eyelids of phlyctenular keratitis patients and healthy volunteers are summarized in Table 2. Mean meiboscores of both the upper and lower lids with phlyctenular keratitis were significantly higher than those of healthy volunteers. In seven of nine patients with unilateral phlyctenular keratitis, we compared the mean meiboscores of inflamed and non-inflamed eyes. In other two patients with unilateral phlyctenular keratitis, the meibomian glands of non-inflamed eyes were not observed. The mean meiboscore of both the upper and lower lids with phlyctenular keratitis (upper lid: 3.0 ± 1.7, lower lid: 2.8 ± 1.4) was significantly higher than that of non-inflamed lids (upper lid: 1.1 ± 1.1, lower lid: 1.1 ± 1.3) (*P* < 0.05). Locations of corneal nodules in phlyctenular keratitis patients were not consistent with the locations of meibomian gland losses. Meibography in patients with phlyctenular keratitis yielded low-contrast images of meibomian glands, due to conjunctival edema, and the loss of distal meibomian glands from meibomian orifices.

## Discussion

In this study, meibography revealed frequent meibomian gland losses in patients with phlyctenular keratitis, while the morphology of meibomian glands in healthy controls was normal. Moreover meiboscore of lids with phlyctenular keratitis was higher than that of non-inflamed lids in patients with unilateral phlyctenular keratitis. Previous studies have reported a relationship between phlyctenular keratitis and inflammation of the eyelids and meibomian glands [3–10]. Koh et al. demonstrated meibomian gland loss in an eye with unilateral marginal staphylococcal keratitis and eyelid inflammation [16]. Meibomian gland inflammation may induce ocular surface inflammatory disease [7]. Morphology of meibomian gland may be influenced by meibomian gland inflammation because inflammatory cells could obstruct meibomian gland. Thus patients with phlyctenular keratitis may accompany with meibomian gland losses. Moreover there are other possibilities. Since abnormalities of meibomian glands have been found in allergic conjunctivitis and in contact lens wearers [13], meibomian gland loss in phlyctenular keratitis might be caused by the mechanical friction between the ocular surface and eyelid, by inflammation of the cornea and conjunctiva, and bacterial exposure. Another possibility is that meibomitis with meibomian gland loss occurs before the appearance of phlyctenular keratitis, and imbalances in the bacterial flora of meibomian glands, including *P. acnes,* induce a corneal DTH reaction to a microbial protein, causing phlyctenular keratitis. However some limitations exist in our study. First, since we did not check bacterial culture of eye lids and meibomian gland in all patients, little is known about relationship between bacteria species and morphology of meibomian gland. Along with *P. acnes*, *Staphylococcus aureus* which can produce toxins and proteases might be related to pathology of meibomian gland. Second, patients group included three adults (>20 years). Thus ages could influence to meibomian gland pathology. We need to increase number of patients and investigate relationship between ages of patients with phlyctenular keratitis and morphology of meibomian gland. Third, we did not check meibography after treatment for phlyctenular keratitis using antibiotics and steroids. It is important to know if morphology of meibomian gland normalizes after disappearance of inflammation in cornea and meibomian gland. Thus further investigation revealing the relationship between meibomian gland loss and phlyctenular keratitis is required.

The diagnosis of phlyctenular keratitis is made based on clinical manifestations, such as corneal nodules, their vascularization, and meibomitis, along with a positive bacterial culture of the eyelid or meibum. Adding to these conventional examinations, noncontact meibography is useful for visualization of the condition of the meibomian glands. Our data demonstrate a high frequency of meibomian gland loss in phlyctenular patients.

## Conclusions

Noncontact meibography enabled visualization of meibomian gland loss in phlyctenular keratitis patients, indicating that meibomian gland abnormality in young individuals may be related to the pathogenesis of phlyctenular keratitis.

## Additional file

Additional file 1: Summary of 13 patients with phlyctenular keratitis. (XLSX 11 kb)

## Abbreviations

DTH: Delayed-type hypersensitivity
MRKC: Meibomitis-related keratoconjunctivitis
SD: Standard deviation
SPK: Superficial punctate keratopathy

## References

[1] Mozayeni RM LS, Krachmer JH MM, Holland EJ (2005). Phylctenular keratoconjunctivitis and marginal staphylococcal keratitis. Cornea fundamentals, diagnosis and management.

[2] Robin JB DR, Robin SB. Immunologic disorders of the cornea and conjunctiva. In: Kaufman HE BB, McDonald MB. Woburn, MA, editors. The cornea, 2nd ed. Philadelphia: Butterworth-Heinemann; 1999: p. 581-2.

[3] Suzuki T, Mitsuishi Y, Sano Y, Yokoi N, Kinoshita S (2005). Phlyctenular keratitis associated with meibomitis in young patients. Am J Ophthalmol.

[4] Smolin G, Okumoto M (1977). Staphylococcal blepharitis. Arch Ophthalmol.

[5] Sorsby A (1942). The aetiology of phlyctenular ophthalmia. Br J Ophthalmol.

[6] Sorsby A (1942). The aetiology of phlyctenular ophthalmia. Br J Ophthalmol.

[7] Suzuki T (2012). Meibomitis-related keratoconjunctivitis: implications and clinical significance of meibomian gland inflammation. Cornea.

[8] Suzuki T, Sano Y, Sasaki O, Kinoshita S (2002). Ocular surface inflammation induced by propionibacterium acnes. Cornea.

[9] Thygeson P (1951). The etiology and treatment of phlyctenular keratoconjunctivitis. Am J Ophthalmol.

[10] Thygeson P (1969). Complications of staphylococcic blepharitis. Am J Ophthalmol.

[11] Nichols JJ, Berntsen DA, Mitchell GL, Nichols KK (2005). An assessment of grading scales for meibography images. Cornea.

[12] Arita R (2013). Validity of noninvasive meibography systems: noncontact meibography equipped with a slit-lamp and a mobile pen-shaped meibograph. Cornea.

[13] Arita R, Itoh K, Maeda S, Maeda K, Amano S (2013). A newly developed noninvasive and mobile pen-shaped meibography system. Cornea.

[14] Shirakawa R, Arita R, Amano S (2013). Meibomian gland morphology in Japanese infants, children, and adults observed using a mobile pen-shaped infrared meibography device. Am J Ophthalmol.

[15] Arita R, Itoh K, Inoue K, Amano S (2008). Noncontact infrared meibography to document age-related changes of the meibomian glands in a normal population. Ophthalmology.

[16] Koh S, Maeda N, Nishida K (2014). Visualization of the meibomian glands by means of noncontact mobile-type meibography (Meibopen) in a 16-year-old girl with unilateral marginal staphylococcal keratitis. J AAPOS.

